# Rank-invariant resampling based estimation of false discovery rate for analysis of small sample microarray data

**DOI:** 10.1186/1471-2105-6-187

**Published:** 2005-07-22

**Authors:** Nitin Jain, HyungJun Cho, Michael O'Connell, Jae K Lee

**Affiliations:** 1 Division of Biostatistics and Epidemiology, Department of Health Evaluation Sciences, University of Virginia School of Medicine, Hospital West Complex, Room. 3181, P.O. Box 800717, Charlottesville, VA 22908-0717, USA; 2 Insightful Corporation, 2505 Meridian Parkway Suite 175, Durham, NC 27713, USA

## Abstract

**Background:**

The evaluation of statistical significance has become a critical process in identifying differentially expressed genes in microarray studies. Classical p-value adjustment methods for multiple comparisons such as family-wise error rate (FWER) have been found to be too conservative in analyzing large-screening microarray data, and the False Discovery Rate (FDR), the expected proportion of false positives among all positives, has been recently suggested as an alternative for controlling false positives. Several statistical approaches have been used to estimate and control FDR, but these may not provide reliable FDR estimation when applied to microarray data sets with a small number of replicates.

**Results:**

We propose a rank-invariant resampling (RIR) based approach to FDR evaluation. Our proposed method generates a biologically relevant null distribution, which maintains similar variability to observed microarray data. We compare the performance of our RIR-based FDR estimation with that of four other popular methods. Our approach outperforms the other methods both in simulated and real microarray data.

**Conclusion:**

We found that the SAM's random shuffling and SPLOSH approaches were liberal and the other two theoretical methods were too conservative while our RIR approach provided more accurate FDR estimation than the other approaches.

## Background

In microarray data analysis, hypotheses relating to differential expression of many genes across the experimental conditions are tested simultaneously. Typical research questions examine the effects of disease status and drug response on the expression of each gene. An extremely large number of e.g. >40K genes can be currently represented on a microarray, so that its statistical results must be carefully analyzed taking a false positive error rate and multiple comparison issues into account. In order to control such a false-positive rate, traditional statistical methods often control the family-wise error rate (FWER), the probability of incorrectly accepting at least one false-positive hypothesis (or type-I error) among all hypotheses; for example, the commonly-used Bonferroni correction divides the type I error *α *by the total number of hypotheses for the test of each gene's differential expression, assuming the hypotheses under consideration are independent [1]. However, this independence assumption is unlikely to be true in microarray data, as functions of many genes are interrelated in varying degrees. Moreover, the methods controlling FWER are frequently found to be too conservative to identify many important genes in biological applications [2]. Several authors (e.g., Sidak, WestFall and Young) have developed step-down procedures that apply the severe Bonferroni correction only to the most extreme value of the test statistic, and step down the correction with the value of the test statistic. However, these methods still result in high false-negative error, likely missing many genes that are truly differentially expressed.

Benjamini and Hochberg (BH) [3] suggested that controlling false discovery rate (FDR), the expected proportion of false positives among all positive (or rejected) hypotheses, is more appropriate for large screening problems. Benjamini and Yekutieli (BY) [4] proposed a new FDR procedure considering a certain dependency structure among the test statistics. However, both the BH and BY procedures may still be too conservative when applied to real microarray data analysis [1]. This is mainly due to the fact that the independence or the artificial dependency assumptions made in these approaches may not be supported in real microarray data applications. Furthermore, microarray experiments are often conducted with a small number of replicates due to limited availability of RNA samples and/or budgetary constraints [2].

**Figure 1:**
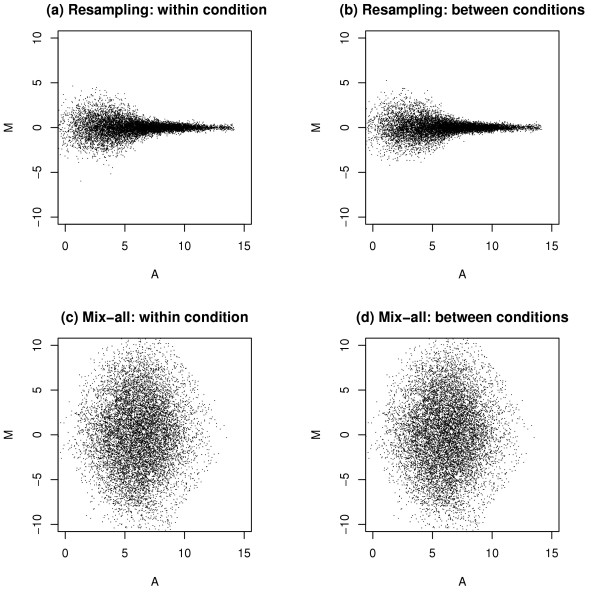
**Scatter plots of null data**. (a) null data within the same condition from the resampling method; (b) null data between the different conditions from the resampling method; (c) null data within the same condition from the Mix-all method; (d) null data between the different conditions from the mix-all method;

One of the key issues in estimating FDR is the assumption regarding the underlying null distribution. The Significance Analysis of Microarrays (SAM) method [5] uses a full permutation strategy, sampling across all genes and conditions to generate such a null distribution (mix-all). However, this strategy breaks many intrinsic correlation structures and does not generate a realistic or *biologically-relevant null *distribution for microarray data (see Figure 1; its detailed explanation in the Result section). Chip-by-chip permutation strategies [1], which randomly shuffle all the columns (chips) and preserve gene structure, are not applicable when the sample size is small because the number of independent permutations is too small to generate a null distribution with enough granularity to support desired significance calculations. In order to provide more stable estimation of such FDR values, a method based on the spacings LOESS histogram (SPLOSH) was also proposed based on a certain assumption about the p-value distribution [6].

In order to overcome these restrictions, we propose a *rank-invariant resampling *(RIR) approach to FDR estimation, especially for microarray data with a small number of replicates. In particular, we use the local pooled error (LPE) test [2], which has high statistical power in analyzing low-replicate microarray data, as a tool for discovery of differential expression. In brief, the LPE approach is based on a model for variance as a function of mean expression intensity, shrinking observed within-gene error estimates by pooling error information of other genes in local intensity ranges and characterizing the variance function by a non-parametric smoother in order to improve the accuracy of error estimation in small sample microarray data analysis. Consequently, the LPE approach provides a dramatically higher statistical power than other *within-gene *test methods, such as SAM and two-sample tests, for identifying differentially expressed genes in microarray data with limited replication. We compare the performance of our approach with that of four other approaches – BH, BY, mix-all, and SPLOSH, using both simulated and real microarray data sets.

## Results

### Simulation study

**Figure 2:**
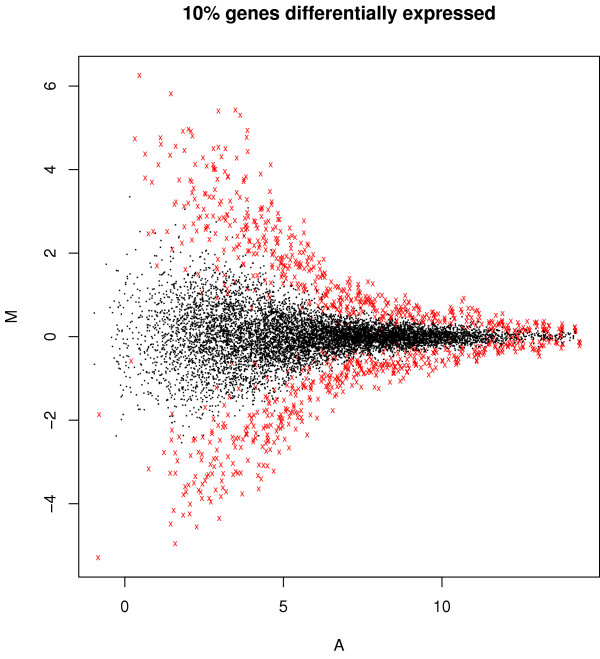
**M vs A plot of simulated data**. The simulated data contains 10% significant genes (indicated by 'x'), and 90% insignificant genes.

We first investigate whether the proposed resampling method provides a realistic null distribution. We generate a set of null data from a real array data set by the proposed resampling method and the mix-all method. Figure 1 displays array-by-array scatter plots of null data from both methods in the from of the so-called A (each gene's average intensity between two arrays) versus M (each gene's intensity difference between two arrays) transformation. First, the scatter plots (a) and (b) by our RIR algorithm show heterogeneous error variances on different intensity ranges assimilating those in the original microarray data quite well. On the other hand, the plots (c) and (d) show much bigger, yet homogeneous error variances regardless of the intensity levels, which are considerably different from those in the real data. For comparing the FDR estimation methods, we generate simulated data as follows. Instead of certain (parametric) distributional assumptions about microarray data, we use real microarray data to obtain such data. That is, let *X*_1 _and *X*_2 _be log2-transformed and normalized data from the replicated chips on the same experimental condition of a microarray study. We first compute M (=*X*_1 _- *X*_2_) and A (=(*X*_1 _+ *X*_2_)/2), and then divide the intensity range of A into 100 intervals. Let  be the maximum of (the absolute value of) M in each interval and Ã is the corresponding A. Then, for equivalently expressed genes, we use means and variances under two different experimental conditions at each interval for our generation of null data. For each of differentially expressed genes, we derive its two means (say *μ*_1 _and *μ*_2_) under two different conditions using equations: (*μ*_1 _+ *μ*_2_)/2 = Ã and (*μ*_1 _- *μ*_2_) = *δ*, where *δ *is a factor determining the degree of differential expression. In this paper, we use it *δ *= 1.5; more discussion about this selection can be found in the Discussion section below. The corresponding variances are obtained from LPE baseline variance estimates. For our simulation study, we generate expression intensities of triplicate arrays with 10,000 genes under each of two conditions with 5%, 10%, 20%, or 50% differentially expressed genes. For example, the Bland-Altman plot (M versus A plot) of a simulated data set with 10% differentially expressed genes is displayed in Figure 2, in which differentially expressed genes are shown in the upper or lower boundaries as points marked with red x's. The above non-parametric, adaptive generation of simulated data has been found to provide the most realistic microarray data and differential expression pattern of many data generation methods and settings tried (data not shown). Note that since our simulated data were randomly generated with the same dynamic ranges and the same underlying resampling distribution, a normalization step was not additionally performed for these simulated data. However, IQR (inter-quartile-range) or non-parametric regression-based normalization (e.g., loess) is recommended prior to the application of the RIR algorithm in practice as in Dudoit et al. (2002).

**Figure 3:**
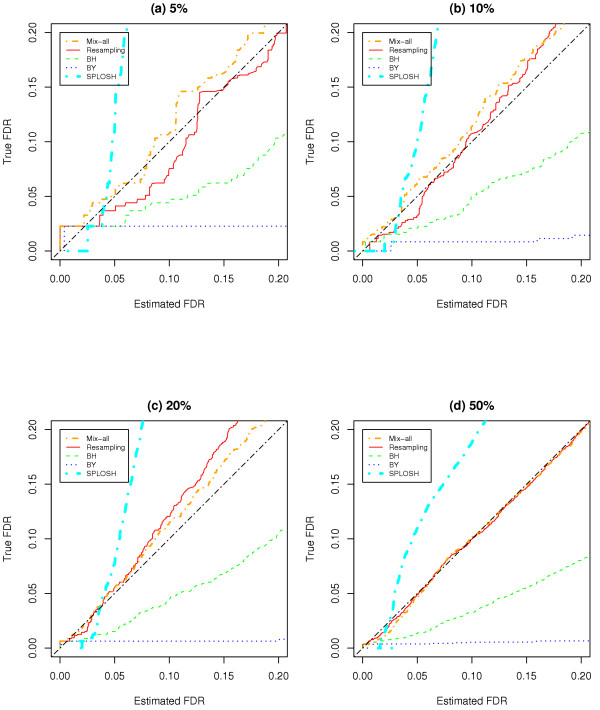
**Comparison of four FDR estimation methods**. (a), (b), (c), and (d) are the plots between true and estimated FDR for simulated data with 5%, 10%, 20%, and 50% differentially expressed genes, respectively.

We then apply LPE to the simulated data sets and estimate FDR by our RIR method, as well as BH, BY, mix-all, and SPLOSH. In brief, using the variance estimates  and  the LPE *z*-statistic is derived as , where  and  are the medians under two conditions and *n*_1 _and *n*_2 _are numbers of replicates in the two experimental conditions being compared; in our simulation study *n*_1 _= *n*_2 _= 3. Next, the FDR levels are estimated with the three FDR evaluation methods. The FDR levels of 0.2 or smaller have been examined because only such levels of FDR would be useful in practice. Figure 3 shows that BH and BY provide very conservative results while the mix-all approach gives somewhat liberal results, especially when a small (less than 10%) percentage of genes are differentially expressed. SPLOSH is conservative at very small FDRs, and then rapidly becomes very liberal. Our RIR method provides the most accurate FDR estimates compared to the others, especially in the cases with a small percentage of differentially expressed genes (5 or 10%).

### Application to the mouse immune response data

The microarray data of the immune response study is used to show performance with real data [2]. This study was performed with triplicate microarrays under each of Naive and 48 hour-activated cells, using Affymetrix MG-U74Av2 chips of 12488 probe sets. Table 1 displays the numbers of the selected differentially expressed genes at FDR 0.0001, 0.001, 0.01, or 0.05. The results again show that BH and BY are more conservative than others, whereas the SPLOSH and mix-all methods are more liberal than the others. Table 2 shows the minimum FDR (or q-value) estimates for the five well-known genes that were reported and confirmed in the original study [2]. The q-value estimates of several genes among them were greater than 0.01 by conservative BH and BY. One or more genes' q-value estimates were greater than 0.01 by SPLOSH and mix-all, whereas RIR identified all of these genes with q-value < 0.01.

**Table 1:** Numbers of differentially expressed genes discovered by five methods

FDR cutoff	BY	BH	SPLOSH	Mix-all	RIR
0.0001	1397	1730	2876	2542	2074
0.001	1730	2162	3134	2958	2485
0.01	2160	2849	3467	3694	3382
0.05	2670	3661	5654	4594	4548

**Table 2:** Minimum FDR estimates of well-known genes found to be differentially regulated genes

Gene Symbol	Gene Title	BY	BH	SPLOSH	Mix-all	RIR
CD97	CD97 antigen	0.0230	0.0023	0.0489	<0.0001	0.0006
GATA3	GATA-binding protein-3	0.0208	0.0021	0.0489	<0.0001	0.0006
Clast3-pending	CD40 ligand-activated specific transcript	0.1005	0.0103	<0.0001	0.0007	0.0034
GZMK	Granzyme K	0.2768	0.0277	0.0524	0.0037	0.0091
FAF1	Fas-associated factor-1	1.0000	0.1100	<0.0001	0.0335	0.0038

## Discussion and Conclusion

In this study we have demonstrated that our RIR-based FDR estimation method significantly outperforms the other popular approaches and provides very accurate FDR estimates, especially when a small percentage of genes are differentially expressed. Among the other FDR evaluation methods compared, the BH and BY methods were found to provide quite conservative results and failed to identify a number of truly differentially expressed genes in real microarray data, whereas the full-permutation (mix-all) approach appeared to yield false positives as significant genes.

In this study we found that one of the most critical steps in FDR evaluation is the generation of biologically-relevant null data. This step has failed and/or is difficult to assimilate in other theoretical and computational FDR estimation approaches. We believe that our heuristic, resampling-based approach provides a significant improvement on FDR estimation and a realistic and intuitive framework for understanding FDR in practice. Other approaches in use are based on quite restrictive mathematical assumptions and/or computational constraints, which result in a biologically unrealistic framework for statistical estimation and discovery. In particular, the simple, full permutation strategy produces both an inflated pooled variance and an inflated difference between the gene intensities, but results in a liberal testing framework because the inflation in the numerator of the test statistics (differential expression) is larger than that in the denominator (variance) in such a null distribution. On the other hand, the shuffling strategies across all conditions can not be applied to microarray data with a small sample size, as the number of independent permutations is too small to provide any meaningful results.

In many microarray studies under controlled experimental conditions, one may expect less than 10% of the genes to be differentially regulated, and thus removal of the top 10% genes from each local interval can be effective in generating a null-distribution excluding most of the differentially expressed genes. Our simulations show that removal of the top 5%, 10%, 20%, or even 50% genes does not affect the null distribution (data not shown), but we admit that these are yet subjective choices and may require a more extensive investigation. Our simulation studies have shown that removing the top 10% of genes produces results close to the true FDR among the four cases with 5% to 50% of differentially expressed genes. In Figure 3, we showed the comparison among the FDR evaluation methods for the simulated data with the proportions of differentially expressed genes varying between 5% and 50%. In many microarray studies, the proportion of differentially expressed genes would be lower than this. Thus, as somewhat expected, the mix-all approach, which is not sensitive to variability across different intensity ranges in microarray data, performs quite well if the proportion of differentially expressed genes is high and a large number of genes do not follow the baseline error distribution. Overall, the bigger such a proportion, the better the mix-all approach would perform. Note that with 5% and 10% of differentially expressed genes, the mix-all method performed poorer, with more liberal, underestimated FDR estimates, than our RIR approach. As Pounds and Cheng [6] reported, the FDR estimates of the mix-all approach are found to be somewhat unstable for low FDR, which may be a critical region in real data applications.

It has often been found that the results from simulation studies may be considerably affected by certain predefined parameters and settings, for example, *δ *for the differential expression magnitude and *q *for the estimation of null-gene proportion in our current study. As such we examined sensitivity of our results to these settings. First, we found that our results were not much different for different choices of *q *between 0.5 – 0.95 (data not shown). Also, although a more reasonable cross-validated approach is yet to be developed for choosing the *δ *value, our current parameter value was empirically chosen from an actual microarray data analysis. We then consistently used this value in our simulation study with varying proportion of differentially expressed genes up to 50% and found little effect of this setting on the resulting null distribution.

We note that our RIR-based FDR estimation is derived for each threshold value *c *of LPE z-score and that the ratio of *V*(*c*) and *R*(*c*) is then calculated only when *R*(*c*) > 0, so that this effectively provides an estimate of *pFDR*(*Z > c*), the q-value. Thus, the RIR-based FDR evaluation can be considered as a carefully designed resampling-based q-value estimation [7]. Note also that our RIR-based approach can be applied to microarray data analysis independent of different preprocessing methods.

In Table 2, several known genes' FDR estimates from the SPLOSH and mix-all approaches were larger than those of RIR. This is somewhat contrary with the observation that the SPLOSH and mix-all approaches were more liberal than the RIR as seen in Fig. 3 and Table 1. This may be due to the fact that these genes have relatively low variability, i.e., in high intensity regions, so that their significance is higher by considering such heterogeneous variability by RIR, but not by the others.

## Methods

### Generation of biologically relevant null distribution

It is critical to generate an underlying null distribution as close as possible to real microarray data because a gene's statistical significance can be dramatically different under different underlying null distributions. Therefore, our resampling strategy is designed to preserve the biological structure of each microarray data set as much as possible. Before describing our resampling strategy, we present an algorithm for constructing intervals, which is used in our resampling strategy. A naive approach for construction of intervals is to partition intensity ranges so that each interval has an equal number of genes. This approach may yield overly large test statistics in high intensity levels because intensities are very sparse in high levels and condense in the middle levels. In order to obtain the local intervals of the genes with homogeneous variances, we therefore construct adaptive intervals by the following algorithm.

### Adaptive Interval (AI) algorithm

1. Estimate a baseline variance function for all data under consideration (within each experimental condition) by LPE

2. Obtain medians and variance estimates for each gene.

3. Order the medians and variances by the medians and denote the ordered medians and variances by *ξ*_(*i*) _and *σ*_(*i*)_.

4. Obtain the first interval with threshold values *ξ*_(1) _and *ξ*_(1) _+ *σ*_(1)_.

5. Obtain the next interval with *ξ*_(2) _and *ξ*_(2) _+ *σ*_(2)_, where *ξ*_(2) _is the smallest median such that *ξ*_(2) _≥ *ξ*_(1) _+ *σ*_(1)_.

6. Repeat step 5 to obtain the next intervals with *ξ*_(*i*) _and *ξ*_(*i*) _+ *σ*_(*i*)_, where *i *is the index of the smallest median such that *ξ*_(*i*) _≥ *ξ*_(*i *- 1) _+ *σ*_(*i *- 1) _until all the data are assigned to certain intervals.

Note that the number of genes in each interval is forced to be between given minimum and maximum numbers. In this paper, we used 10 and (1/100 of the total number of genes) for the minimum and maximum numbers, respectively. Note also that this AI algorithm is applied to the replicated array data under each experimental condition separately.

Our RIR procedure for generating null data is then as follows.

1. Calculate medians for each gene and obtain the ranks of these medians within each experimental condition.

2. Calculate rank differences between two conditions for each gene.

3. Construct the first intensity intervals using the AI algorithm above and retain rank-invariant genes by eliminating a certain percentage of genes with largest rank differences within each interval.

4. Construct the final intensity intervals of rank-invariant genes using the AI algorithm.

5. Obtain a set of null data by resampling intensities of rank-invariant genes within each interval.

6. Repeat the above step B times, e.g., 1,000, to obtain B independent sets of resampled null data.

In step 5 of the above procedure, a certain percentage of genes are eliminated to retain only rank-invariant expressed genes. In this current application, we remove 50% of all genes with largest rank differences; a discussion regarding other choices is presented later. Note that the AI algorithm is used twice in this RIR procedure; the first time to remove rank-variant genes evenly throughout the whole intensity ranges. Without this step, it was found that many genes in low intensity ranges were unproportionately removed due to the larger variability in those ranges (data not shown). This is a particularly important issue for Affymetrix data that have been summarized using the MAS5 procedure.

### Estimation of FDR based on the RIR procedure

We calculate LPE Z-statistics *Z*_*null *_from null data as generated following the procedure described above. Generation of the null data is repeated many times independently. Let *Z*_*real *_be a LPE Z-statistic computed from the real data. FDR at a threshold value *c *can be estimated as



where *V*(*c*) is the average number of *Z*_*null *_equal to or greater than *c *and *R*(*c*) is the number of *Z*_*real *_equal to or greater than *c*. The proportion *π*_0 _of true null genes in real data can be estimated by the number of {*Z*_*real *_≤ *λ*_*q*_} divided by the average number of {*Z*_*null *_≤ *λ*_*q*_}, where *λ*_*q *_is the *q*-th quantile of *Z*_*null *_as suggested by Storey and Tibshirani (2003). In this paper, we use 0.9 for *q*; more discussion about this choice can be found in the Discussion section below. A gene's FDR value might be estimated as zero when no gene in the resampled null data exceeds its *Z*_*real*_; in these cases we force the minimum estimate of FDR to be the reciprocal of the product between the numbers of genes and resampled null data sets, which is the finest resolution of our RIR FDR estimation. Note that the confidence bounds for  at each threshold value *c *can also be obtained from the B resampled null data sets.

### Other FDR estimation methods

SAM's full permutation (or *mix-all*) strategy randomly samples all intensity values across genes and conditions to generate null data, of which FDR estimation can be similarly performed as described above for our RIR approach. Benjamini and Hochberg (BH) [3] proposed the step-up procedure to control FDR. These approaches can be compared with our RIR approach based on the LPE statistics in the following manner. Let *z*_(1) _≥ *z*_(2) _≥ ... ≥ *z*_(*G*) _be LPE *z*-statistics for discovery of differential expression of *G *genes. Denote the corresponding ordered raw *p*-values as *p*_(1) _≤ *p*_(2) _≤ ... ≤ *p*_(*G*)_. BH adjusted *p*-values are defined as  = min_*k*=*i*,...,*G*_{min(*p*_(*k*)_*G/k*, 1))}. For control of FDR at level *α*, a gene *i *is claimed as significant if  ≤ *α*. Thus, the BH estimate of FDR at a given critical value *c *can conservatively be defined as , where i* is min{*i *: *z*_(*i*) _≥ *c*}. The adjusted *p*-values of Benjamini and Yekutieli (BY) [4] are defined as . Utilizing the information in both left-hand and right-hand sides of the p-value distribution, the SPLOSH FDR estimate is *h*_(*i*) _= min_*k*≥*i*_(*r*_(*k*)_), where *r*_(*k*) _is a conditional FDR (cFDR) estimate of gene *k *and cFDR is a FDR given the number of positives [8]. These four methods for FDR estimation are compared with our RIR method in the next section.

## Authors' contributions

N.J. wrote the computer code and did the simulation work. All the authors contributed in developing the idea and wrote the manuscript.
